# Editorial of Special Issue: Biological Basis of Musculoskeletal Regeneration 2019

**DOI:** 10.3390/ijms21175968

**Published:** 2020-08-19

**Authors:** Franka Klatte-Schulz, Britt Wildemann

**Affiliations:** 1 Julius Wolff Institute, BIH Center for Regenerative Therapies, Charité—Universitätsmedizin Berlin, Corporate Member of Freie Universität Berlin, Humboldt-Universität zu Berlin, and Berlin Institute of Health, 13353 Berlin, Germany; franka.klatte@charite.de; 2 Experimental Trauma Surgery, Department of Trauma, Hand and Reconstructive Surgery, University Hospital Jena, 07747 Jena, Germany

The Special Issue “Biological Basis of Musculoskeletal Regeneration 2019” aimed to collect research and review articles that cover various aspects of the molecular and cellular mechanisms of bone, cartilage, tendon/ligament, and muscle regeneration. The different tissues of the musculoskeletal system are very heterogeneous and range from hard to soft tissues, with good or limited healing potential, but have in common that optimal regeneration is necessary for proper function. The current research addresses the biological processes responsible for regeneration or aims to develop treatment strategies to promote regeneration. The in vitro and in vivo studies of this Special Issue cover different aspects of musculoskeletal regeneration regarding biomaterials, tissue engineering, genetics, cell modifications and animal models. A total of 10 original papers and two review articles are published, as summarized in Table 1.

## Publications of The Special Issue 2019

Lauer, A.; Wolf, P.; Mehler, D.; Götz, H.; Rüzgar, M.; Baranowski, A.; Henrich, D.; Rommens, P.; Ritz, U. Biofabrication of SDF-1 Functionalized 3D-Printed Cell-Free Scaffolds for Bone Tissue Regeneration. *Int. J. Mol. Sci.* **2020**, *21*, 2175, doi:10.3390/ijms21062175.
Westhauser, F.; Hohenbild, F.; Arango-Ospina, M.; Schmitz, S.; Wilkesmann, S.; Hupa, L.; Moghaddam, A.; Boccaccini, A. Bioactive Glass (BG) ICIE16 Shows Promising Osteogenic Properties Compared to Crystallized 45S5-BG. *Int. J. Mol. Sci.* **2020**, *21*, 1639, doi:10.3390/ijms21051639.
Altinbas, L.; Bormann, N.; Lehmann, D.; Jeuthe, S.; Wulsten, D.; Kornak, U.; Robinson, P.; Wildemann, B.; Kararigas, G. Assessment of Bones Deficient in Fibrillin-1 Microfibrils Reveals Pronounced Sex Differences. *Int. J. Mol. Sci.* **2019**, *20*, 6059, doi:10.3390/ijms20236059.
Otto, E.; Knapstein, P.R.; Jahn, D.; Appelt, J.; Frosch, K.H.; Tsitsilonis, S.; Keller, J. Crosstalk of Brain and Bone-Clinical Observations and Their Molecular Bases. *Int. J. Mol. Sci.* **2020**, *21*, 4946, doi:10.3390/ijms21144946.
Schwarz, S.; Gögele, C.; Ondruschka, B.; Hammer, N.; Kohl, B.; Schulze-Tanzil, G. Migrating Myofibroblastic Iliotibial Band-Derived Fibroblasts Represent a Promising Cell Source for Ligament Reconstruction. *Int. J. Mol. Sci.* **2019**, *20*, 1972, doi:10.3390/ijms20081972.
Sauerschnig, M.; Berninger, M.; Kaltenhauser, T.; Plecko, M.; Wexel, G.; Schönfelder, M.; Wienerroither, V.; Imhoff, A.; Schöttle, P.; Rosado Balmayor, E.; Salzmann, G. Chondrocyte Culture Parameters for Matrix-Assisted Autologous Chondrocyte Implantation Affect Catabolism and Inflammation in a Rabbit Model. *Int. J. Mol. Sci.* **2019**, *20*, 1545, doi:10.3390/ijms20071545.
Riedl, M.; Witzmann, C.; Koch, M.; Lang, S.; Kerschbaum, M.; Baumann, F.; Krutsch, W.; Docheva, D.; Alt, V.; Pfeifer, C. Attenuation of Hypertrophy in Human MSCs via Treatment with a Retinoic Acid Receptor Inverse Agonist. *Int. J. Mol. Sci.* **2020**, *21*, 1444, doi:10.3390/ijms21041444.
Stich, S.; Jagielski, M.; Fleischmann, A.; Meier, C.; Bussmann, P.; Kohl, B.; Schmidt, J.; Krüger, J.; Endres, M.; Cabraja, M.; Reimann, K.; Laue, D.; Ertel, W.; Sittinger, M. Degeneration of Lumbar Intervertebral Discs: Characterization of Anulus Fibrosus Tissue and Cells of Different Degeneration Grades. *Int. J. Mol. Sci.* **2020**, *21*, 2165, doi:10.3390/ijms21062165.
Langendorf, E.; Rommens, P.; Drees, P.; Mattyasovszky, S.; Ritz, U. Detecting the Effects of the Glucocorticoid Dexamethasone on Primary Human Skeletal Muscle Cells—Differences to the Murine Cell Line. *Int. J. Mol. Sci.* **2020**, *21*, 2497, doi:10.3390/ijms21072497.
Haddouti, E.; Randau, T.; Hilgers, C.; Masson, W.; Walgenbach, K.; Pflugmacher, R.; Burger, C.; Gravius, S.; Schildberg, F. Characterization and Comparison of Human and Ovine Mesenchymal Stromal Cells from Three Corresponding Sources. *Int. J. Mol. Sci.* **2020**, *21*, 2310, doi:10.3390/ijms21072310.
Walter, S.G.; Randau, T.M.; Hilgers, C.; Haddouti, E.M.; Masson, W.; Gravius, S.; Burger, C.; Wirtz, D.C.; Schildberg, F.A. Molecular and Functional Phenotypes of Human Bone Marrow-Derived Mesenchymal Stromal Cells Depend on Harvesting Techniques. *Int. J. Mol. Sci.* **2020**, *21*, 4382, doi:10.3390/ijms21124382.
Yamaguchi, F.S.M.; Shams, S.; Silva, E.A.; Stilhano, R.S. PRP and BMAC for Musculoskeletal Conditions via Biomaterial Carriers. *Int. J. Mol. Sci.* **2019**, *20*, 5328, doi:10.3390/ijms20215328.

## Figures and Tables

**Table 1:** Summary of the articles published in the Special Issue.

Tissue/Cells	Authors	Main Message
***Bone***	Lauer, A.; Wolf, P.; Mehler, D.; Götz, H.; Rüzgar, M.; Baranowski, A.; Henrich, D.; Rommens, P.; Ritz, U.	The regeneration of large bony defects is still challenging. Optimized biomaterial functionalized with SDF-1 revealed osteoinductive character and might support regeneration. *In vitro* and *in vivo* studies
	Westhauser, F.; Hohenbild, F.; Arango-Ospina, M.; Schmitz, S.; Wilkesmann, S.; Hupa, L.; Moghaddam, A.; Boccaccini, A.	Bioactive glass is a promising material for bone regeneration. Modification of the chemistry influences the biological effect. The presented bioactive glass modification promoted the osteogenic differentiation of mesenchymal stromal cells (MSC). *In vitro* studies
	Altinbas, L.; Bormann, N.; Lehmann, D.; Jeuthe, S.; Wulsten, D.; Kornak, U.; Robinson, P.; Wildemann, B.; Kararigas, G.	Marfan syndrome is a genetic defect of the connective tissue and a mutation of fibrillin-1, which also affects musculoskeletal tissues. The study investigated possible differences in bone microarchitecture, mechanical properties and TGF-β1 between male and female mice lacking fibrillin-1. *In vivo* studies
	Otto, E.; Knapstein, P.R.; Jahn, D.; Appelt, J.; Frosch, K.H.; Tsitsilonis, S.; Keller, J.	Clinical evidence indicates a bidirectional communication between brain and bone. This review summarizes the existing knowledge on brain–bone communication but presents also data for a bone–brain communication. Review
***Ligament***	Schwarz, S.; Gögele, C.; Ondruschka, B.; Hammer, N.; Kohl, B.; Schulze-Tanzil, G.	Allografts are still the gold standard for the repair of anterior cruciate ligament (ACL) ruptures. This comparative analysis of cells from the iliotibial band (ITB) and the ACL showed the suitability of the ITB cells for ACL reconstruction and possible tissue engineering approaches. *In vitro* studies
***Cartilage***	Sauerschnig, M.; Berninger, M.; Kaltenhauser, T.; Plecko, M.; Wexel, G.; Schönfelder, M.; Wienerroither, V.; Imhoff, A.; Schöttle, P.; Rosado Balmayor, E.; Salzmann, G	One option in the treatment of large cartilage defects is the use of autologous chondrocyte transplantations. Cell culturing conditions might affect the in vivo performance of transplanted cells. The authors showed that the expression of inflammatory and matrix remodeling factors by chondrocytes used for autologous chondrocyte implantation was influenced by culturing conditions. In vitro and in vivo studies
	Riedl, M.; Witzmann, C.; Koch, M.; Lang, S.; Kerschbaum, M.; Baumann, F.; Krutsch, W.; Docheva, D.; Alt, V.; Pfeifer, C.	Chondrocyte hypertrophy is unwanted in cartilage tissue engineering and was reduced by the treatment of MSCs with a retinoic acid receptor inverse agonist. *In vitro* studies.
***Annulus Fibrosus***	Stich, S.; Jagielski, M.; Fleischmann, A.; Meier, C.; Bussmann, P.; Kohl, B.; Schmidt, J.; Krüger, J.; Endres, M.; Cabraja, M.; Reimann, K.; Laue, D.; Ertel, W.; Sittinger, M.	Back pain is a frequent musculoskeletal disorder and can be caused by degeneration of the intervertebral disk. The present study demonstrated differences of anulus fibrosus (AF) cells depending on AF degeneration, which might impact tissue engineering strategies. *In vitro* studies
***Muscle***	Langendorf, E.; Rommens, P.; Drees, P.; Mattyasovszky, S.; Ritz, U.	Back pain can also be caused by muscle atrophy, which can be a side effect of long-term glucocorticoid treatment. The analysis of human skeletal muscle cells revealed a time and concentration-dependent effect of glucocorticoids, but the differentiation status of the cells was also important. *In vitro* studies
***Stromal cells***	Haddouti, E.; Randau, T.; Hilgers, C.; Masson, W.; Walgenbach, K.; Pflugmacher, R.; Burger, C.; Gravius, S.; Schildberg, F.	Sheep are often used in musculoskeletal research and the direct comparison of ovine and human MSCs revealed a good comparability promoting sheep as a reliable preclinical animal model. *In vitro* studies
	Walter, S.G.; Randau, T.M.; Hilgers, C.; Haddouti, E.M.; Masson, W.; Gravius, S.; Burger, C.; Wirtz, D.C.; Schildberg, F.A.	The study found differences in human bone marrow-derived MSCs taken from the same tissue and donor site but harvested either as aspirate or as bone chip. The results indicate that a standardization of the harvesting method might be important. *In vitro* studies
***PRP/BMAC***	Yamaguchi, F.S.M.; Shams, S.; Silva, E.A.; Stilhano, R.S.	New biological treatment strategies for musculoskeletal regeneration are needed. Platelet-rich plasma and bone marrow aspirate concentrate are used and their effectivity might be improved by combining them with biomaterials. Review

